# Investigation of the Structure Requirement for 5-HT_6_ Binding Affinity of Arylsulfonyl Derivatives: A Computational Study

**DOI:** 10.3390/ijms12085011

**Published:** 2011-08-08

**Authors:** Ming Hao, Yan Li, Hanqing Li, Shuwei Zhang

**Affiliations:** Department of Materials Science and Chemical Engineering, Dalian University of Technology, Dalian 116023, Liaoning, China; E-Mails: dluthm@yeah.net (M.H.); lihanqing00@gmail.com (H.L.); zswei@dlut.edu.cn (S.Z.)

**Keywords:** 5-HT_6_, 3D-QSAR, CoMFA, CoMSIA, molecular dynamics

## Abstract

5-HT_6_ receptor has been implicated in a series of diseases including anxiety, depression, schizophrenia and cognitive dysfunctions. 5-HT_6_ ligands have been reported to play a significant role in the treatment for central nervous system (CNS) diseases. Presently, a large series of 223 5-HT_6_ ligands were studied using a combinational method by 3D-QSAR, molecular docking and molecular dynamics calculations for further improvement of potency. The optimal 3D models exhibit satisfying statistical results with *r*^2^_ncv_, *q*^2^ values of 0.85 and 0.50 for CoMFA, 0.81 and 0.53 for CoMSIA, respectively. Their predictive powers were validated by external test set, showing *r*^2^_pred_ of 0.71 and 0.76. The contour maps also provide a visual representation of contributions of steric, electrostatic, hydrophobic and hydrogen bond fields as well as the prospective binding models. In addition, the agreement between 3D-QSAR, molecular docking and molecular dynamics simulation proves the rationality of the developed models. These results, we hope, may be helpful in designing novel and potential 5-HT_6_ ligands.

## 1. Introduction

Nowadays, several central nervous system (CNS) diseases such as cognitive dysfunctions, anxiety and depression are widespread and sometimes even fatal, which leads to both medical and social challenges. Some chemical agents, such as Ro 04-6790, SB-271046, MS-245 and so on currently have been used in Phase I and II clinical trials for cognitive impairment [1–3]. However, there are still some factors like the side effects that prohibit the widespread use of these medicinal therapies. The discovery of serotonin receptor 5-HT_6_ brings new approaches to deal with this kind of mood diseases. Being expressed in the brain regions as known to be associated with learning and memory [4], the 5-HT_6_ receptor plays a role in both the cognitive processes and mood control, as well as in depression and anxiety [5–7]. As a matter of fact, the 5-HT_6_ receptor has emerged as a very interesting molecular target that interacts with both antidepressant and anxiolytic drugs [8].

The rat 5-HT_6_ receptor was first identified in 1993 and thereafter, human 5-HT_6_ gene was cloned and characterized by Kohen *et al.* in 1996 [9]. Consisting of 440 amino acids, human 5-HT6 has seven hydrophobic regions sufficient to span the membrane, which places the receptor in the G-protein-coupled, with seven putative transmembrane domains, receptor superfamily.

5-HT_6_ receptor is mainly found in the central nervous system and ultrastructural studies suggest that they mediate the postsynaptic, rather than the presynaptic actions [9–11]. To clarify the role of 5-HT_6_ receptor in the central nervous system, currently, more and more potent 5-HT_6_ ligands were synthesized such as MS-245 [12], 4′-amino derivative of MS-245 [13], a series of ligands synthesized at Wyeth Research, most of which comprised of heterocyclic core with a basic amine and an arylsulfonyl moiety [14–21] and others [2,22–24]. Several studies indicate that 5-HT_6_ ligands may be cognition enhancers; however, not all studies have come up with the similar result [25]. Therefore, the development of additional potent and selective 5-HT_6_ ligands will provide additional tools for delineating the role of the 5-HT_6_ receptor.

At present, computational applications, such as the quantitative structure-activity relationship (QSAR), pharmacophore [26,27] and molecular dynamics (MD) [28,29], have been widely used in modern drug design aiding the exploration of drug-receptor interaction. These models are able to either reveal the mechanism of drug-receptor interactions or/and predict the biological activity of compounds by their structural properties, usually being of great help for the design of novel potent molecules. For the development of new potent 5-HT_6_ receptor ligands, it’s meaningful to explore the impacts of various substituents on the biological activity of the chemicals. Up to now, to our best knowledge, only several computational studies were performed concerning with the 5-HT_6_ receptor-ligand interactions. In 2004, a three-dimensional quantitative structure activity relationship (3D-QSAR) study which contained 33 antagonists with *K*_i_ values ranging from 1.3 to 1700 nM was reported by Doddareddy M.R *et al.* [30]. Following that, a three-dimensional pharmacophore model for 5-HT_6_ receptor antagonists was built based on forty-five structurally diverse 5-HT_6_ receptor antagonists by Campillo, *etc.* [31]. In that paper, several import pharmacophore features have been identified to interact with the modeled 5-HT_6_ receptor. Besides, two 2D-QSAR models recently were also successfully developed based on relative small number of 5-HT_6_ receptor ligands [32,33].

Thus in the present work, based on a more diverse set of 223 5-HT_6_ ligands, various of *in-silico* models in combination use of 3D-QSAR, molecular docking and molecular dynamics were carried out to find the stereo-electronic parameters with principal aim to render assistance to the development of new 5-HT_6_ receptor ligands.

## 2. Results and Discussion

To measure the predictive capability of a QSAR model, several statistical parameters including especially the cross-validated correlation coefficient (*q*^2^), non-cross-validated correlation coefficient (*r*^2^_ncv_), and standard error of estimate (*SEE*), *F*-statistic values, predicted correlation coefficient for the test set of compounds (*r*^2^_pred_) as well as the optimum number of components (*OPN*) were taken into consideration.

### 2.1. 3D-QSAR Statistical Results

Among the QSAR investigations, two factors are crucial that they almost determine the quality of the model established. One is the molecular descriptors that are used to extract the structural information, which serve as a bridge correlating the structures of the molecules being studied with their biological activities, either in the form of numerical or digital representations. In the present work, two 3D descriptors for CoMFA (*i.e.*, the steric and electrostatic fields) and five 3D descriptors for CoMSIA (*i.e.*, the steric, electrostatic, hydrophobic, hydrogen bond (H-bond) donor and acceptor fields) were applied as the independent variables in the building models. However, though with all possible 31 combinations of these field descriptors applied on the dataset, no CoMFA or CoMSIA models with satisfying statistical results could be obtained (data not shown). Thus, the help of appropriate 2D descriptors is a necessity to the successful modeling of the dataset. During this process, two 2D parameters which are most relevant to the binding affinity of the 5-HT_6_ ligands, *i.e.*, CIC2 and BEHv2, were employed in the model development. Their relevance to the activity was determined by the following process: (1) Originally, 685 two-dimensional parameters were calculated for each molecule by Dragon (DRAGON Professional version 5.4), a commercial soft package which has been applied in many successful QSAR analyses. The variables describe the structural features of the molecules from various angles including the constitutional and topological aspects, walk and path counts, connectivity and edge adjacency indices, Burden eigenvalues, topological charge and eigenvalue-based indices, functional group counts, atom-centred fragments and molecular properties, *etc*.; (2) The correlation of these parameters with the 5-HT_6_ binding affinity was then explored by the multiple linear regression analysis (MLR), ending up in a model with several variables as the most relevant ones to the biological activity, where the first two crucial ones (CIC2 and BEHv2) were selected to aid in the building of 3D-QSAR models.

The second factor impacting greatly on the model quality is the appropriate superimposition of all molecules prior to the PLS analysis. Since up to now the X-ray crystal structure of 5-HT_6_ receptor is still unavailable, homology modeling for the protein was still required presently. Thus in this work, both the ligand- and receptor-based alignment rules were applied, with purpose to compare and implement the results of them for exploring the receptor-ligand interaction mechanism as real as possible. As a result, several optimal CoMFA and CoMSIA models with proper predictive performance based on the same training (184 molecules) and test set (39 molecules) were obtained, with their statistical results shown in Table 1. Clearly, both the two 2D descriptors of CIC2, which is an information index describing the complementary information content (neighborhood symmetry of 2-order) and BEHv2, the highest eigenvalue n. 2 of Burden matrix weighted by atomic van der Waals volumes Burden eigenvalues, display their little, but critical contribution to the models.

For the ligand-based study, the optimal CoMFA model employing both the steric and electrostatic field descriptors obtains a LOO cross-validated *q*^2^ of 0.50, a correlation coefficient *r*^2^_ncv_ of 0.85, a *SEE* value of 0.27 and an *F* value of 127.90 using 7 components, indicating a good internal predictivity of the model. When being validated by the independent test set which is not applied in the building of the model, an *r*^2^_pred_ = 0.71 is achieved, proving its reliable external predictivity. As to the field contribution, the steric and electrostatic field descriptors account for 0.42 and 0.46, respectively, to the CoMFA model.

During the ligand-based CoMSIA analysis, a model with better statistical results (*q*^2^ = 0.53, *r*^2^_ncv_ = 0.81, *SEE* = 0.30, *F* = 104.23) than the CoMFA was built, with four field (of steric, electrostatic, hydrophobic and hydrogen bond acceptor) descriptors employed along with CIC2 and BEHv2. Particularly, the *r*^2^_pred_ for the 39 test set compounds is as high as 0.76, which is very close to the *r*^2^_ncv_ of the 184 training set ligands, proving the better external predictive capability. In term of the relative contribution, the electrostatic field makes the greatest (0.33) to the CoMSIA model, followed by hydrophobic field (0.24), then the steric and hydrogen bond acceptor fields give 0.18 and 0.12, respectively.

For the receptor-based 3D-QSAR studies, two optimal models with a little worse but still acceptable statistical results were obtained taking into account of the large number and big structural diversity of the molecules, where the CoMFA model exhibits *q*^2^ = 0.45, *r*^2^_ncv_ = 0.78, *r*^2^_pred_ = 0.59, and the CoMSIA one *q*^2^ = 0.47, *r*^2^_ncv_ = 0.75, *r*^2^_pred_ = 0.62, respectively. Several phenomena similar to the ligand-based models are observed in the two receptor-based ones, which are: (1) Both the two 2D descriptors of CIC2 and BEHv2 are involved in the model development, and their contribution are, though not much, crucial to the models, as demonstrated by their sum as 0.12 and 0.13 in the ligand-based, and 0.16 and 0.17 in the receptor-based CoMFA and CoMSIA models, respectively; (2) Steric and electrostatic interactions display more important and almost the same contributions to the CoMFA models, which are 0.42, 0.46 in the ligand-based, and 0.42, 0.42 in the receptor-based ones, respectively. As for the CoMSIA models, their roles are also shown by the 0.18, 0.33 in ligand-based and 0.16, 0.26 in the receptor-based ones, respectively; (3) The contribution of two other field descriptors, *i.e.*, the hydrophobic and H-bond acceptor, also come into attention that they account for 0.24, 0.12 in ligand-based and 0.25, 0.16 in receptor-based CoMSIA models, respectively. When compared with each other, all CoMSIA models exhibit better performance than their corresponding CoMFA ones, which may be probably due to the incorporation of the two additional field descriptors. This, from anther aspect, may indicate the importance of hydrophobic and H-bond interactions for the 5-HT_6_ receptor and ligands.

**Figure 1.**
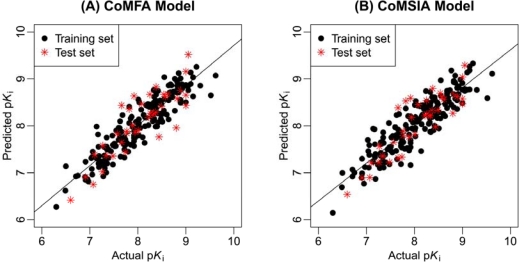
The correlation plots of the predicted versus the actual p*K*_i_ values using the training set (black dot) and the test set (red asterisk) based on the (**A**) CoMFA and (**B**) CoMSIA of ligand-based model.

As shown in Table 1, since the ligand-based model gives the optimal results, we just limit our further research to this model. Figure 1 illustrates the correlation plots of the experimental versus the predicted p*K*_i_ values of the training (black dot) and test (red asterisk) sets for both the ligand-based 3D-QSAR models. Clearly, good correlationships are observed since the predicted values are almost as accurate as the experimental activities for the whole dataset. Table S1 (Supporting Information) lists the predicted results of the whole data set.

### 2.2. Contour Maps

**Figure 2.**
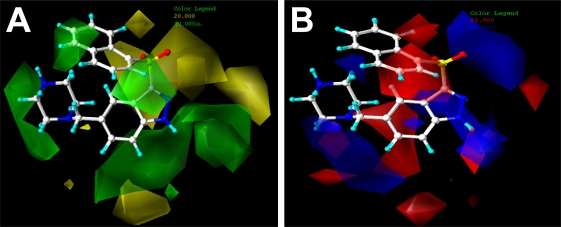
CoMFA StDev*Coeff contour plots for 5-HT_6_ receptor ligands in combination with compound 198. (**A**) Steric (green/yellow) contour map. Green contours indicate regions where bulky groups increase activity (favored level 80%); yellow contours indicate regions where bulky groups decrease activity (disfavored level 20%); (**B**) Electrostatic contour map (blue/red). Blue contours indicate regions where positive charges increase activity (favored level 80%); red contours indicate regions where negative charges increase activity (disfavored level 20%).

**Figure 3.**
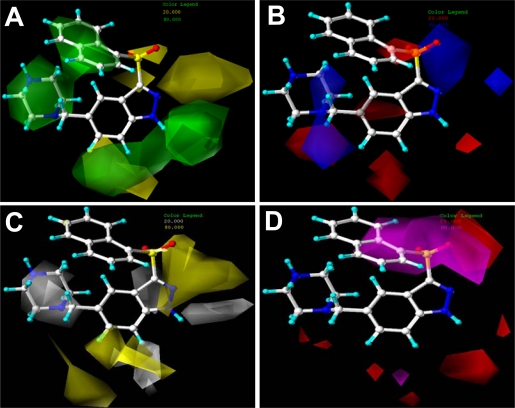
Ligand-based CoMSIA StDev*Coeff contour plots combined with compound 132. (**A**) Steric (green/yellow) contour map. Green contours indicate regions where bulky groups increase activity (favored level 80%); yellow contours indicate regions where bulky groups decrease activity (disfavored level 20%); (**B**) Electrostatic contour map (blue/red). Blue contours indicate regions where positive charges increase activity (favored level 80%); red contours indicate regions where negative charges increase activity (disfavored level 20%); (**C**) Hydrophobic contour map (yellow/white). Yellow contours indicate regions where hydrophobic substituents enhance activity (favored level 80%); white contours indicate regions where hydrophobic substituents decrease activity (disfavored level 20%); (**D**) CoMSIA contour maps illustrating hydrogen-bond acceptor features (magenta/red). The magenta contour indicates regions where H-bond acceptor groups increase activity (favored level 80%); red contour indicates the disfavored region for H-bond acceptor groups (disfavored level 20%).

The CoMFA and CoMSIA results are usually represented as 3D coefficient contour maps which show regions where variations of different field in the structural features of the molecules cause the increase or decrease of the activity. In this study the most potent compound 198 is shown as an example molecule in all the following CoMFA and CoMSIA contour maps (Figures 2 and 3).

The steric and electrostatic fields from the best CoMFA model are represented in Figure 2. In the steric field (Figure 2A), the green colored contours represents regions of favorable steric effect, while yellow colored contours represent regions of unfavorable steric effect, respectively.

As shown in Figure 2A, there exists a large green contour at the distal of the piperazine ring linked to the 5-position on the indazole, indicating the presence of a bulky group in this position will increase the binding affinity of the class of compounds. The observation can be supported by the experimental results. For example, a comparison among 4-piperidinylamino indazoles (compounds 179, 185, 189, 193 shown in Supporting Information, Table S2) comes to a conclusion that the large substituent at 5-position on the indazole keeps optimal, since in this position the piperazine ring fall into the green-colored zone. In addition one can notice that both 3-piperidinylamino indazoles (compounds 180, 186, 190 and 194 shown in Table S2) and 4-piperidinylmethylamino indazoles (compounds 181, 187, 191 and 195 shown in Table S2) series also provide the same trend that substituents at 5-postion illustrate much bigger (actually, the biggest) potency, as compared to other positions. It can also be found by inspecting the steric contour map that the 4-substituents only show slightly less potency than 5-position. This is consistent with the steric contour map where the 4-position substituent is directing to another green-colored map close to the terminal part of the naphthyl substituent (Figure 2A). On the contrary, the 6- and 7-positions are significantly less potent in affinity than 5-position. This is because that substituent at 6- or 7-postion is connected to the yellow-colored area which is disfavored in a bulk substituent. For 3-sulfonylindazoles (compounds 199–213) classes, we come to the same conclusion that derivatives substituted at 5-position are optimal among ligands 199, 201 and 206. Also it is the same case for compounds 200, 205 and 210.

Two green-colored polyhedra also appear around the naphthyl group, showing that large substituents are likely to enhance the affinity of the ligand. The fact that compound 185 presents larger potency, while its counterpart 214 to substitute the naphthyl group with a relatively small benzene ring shows less activity is just the case. A band of green maps are observed on the lower right of the indazole ring indicating that the substructure fragments with steric bulk in this area increase the activity. This is also consistent with the experimental results that compound 108 with 1-Naph shows high activity (p*K*_i_ = 8.51) due to its large –Naph group that falls into the green map. While its counterparts 99–107 (arylsulfonyl derivatives at the 4-position shown in Table S2) with relatively small substituents give the less potency.

Several yellow-colored maps around the sulfonyl group at the 3-position of indazole suggest that substructure with large groups tend to reduce the activity. The probable reason is that large substitents linked to this position will collide with the surrounding amino acid residues, yielding a negative role in affinity activity.

The CoMFA electrostatic contour map with the representative compound 198 is shown in Figure 2B. Blue contours mean that positive charged substituents are helpful for activity while red contours indicate that negative charges are conducive. As shown in Figure 2B, blue contour maps are observed surrounding the piperazine ring and –NH of indazole group, suggesting that a charge withdrawing group fixed to these positions will enhance the biological activity. In addition, red contour maps exist around the sulfonyl group, thus in this position electro-rich groups are beneficial for increasing the activity. This may be a reason that all compounds of the dataset contain such electronegative groups.

The CoMSIA steric and electrostatic contour maps (Figure 3A, B) are similar to above CoMFA model and is thus not discussed here additionally. Figure 3C shows the CoMSIA hydrophobic field contour map, where yellow contours indicate hydrophobic favorable regions, while white contours illustrate regions where hydrophobic substructures are likely to decrease the activity. One can see that a white contour is embedding the piperazine ring of compound 198, which indicates that in this zone the hydrophobic group is disfavored to affinity activity. Also there exist some white maps near the –NH group of the indazole. Thus an introduction of hydrophilic substituent may be beneficial to the potency. Besides, some yellow-colored polyhedra are observed clamping the indazole ring, indicating that hydrophobic groups such as the benzene ring play a positive role in the activity increase. In the studied compounds, most ligands contain such indazole or other heterocyclic cores as a template. It seems that it is such hydrophobic groups clamped in the middle of hydrophobic favored contours that may hold the ligand in the necessary active orientation.

Figure 3D depicts the H-bond acceptor contour map of the CoMSIA model. Magenta contours encompass areas where an H-bond acceptor will lead to improved biological activity, while an acceptor located near the red regions will result in the loss of biological activity. In this dataset, it can be easily found that a large magenta-colored map is surrounding the –SO_2_ group, which indicates that the presence of H-bond acceptor groups in this region will enhance the affinity. Many compounds of this class show high activity is just due to the –SO_2_ group they possess linked to the indazole at the 3-position as H-bond acceptors. There also exist red-colored maps near the –NH group on the indazole indicating H-bond acceptors in the zone are not beneficial to increase the activity. Thus this information obtained from the CoMFA and CoMSIA contour maps are helpful for us to understand the hypothetical interaction features of the 5-HT_6_ ligands.

### 2.3. Homology Modeling Results

**Figure 4.**
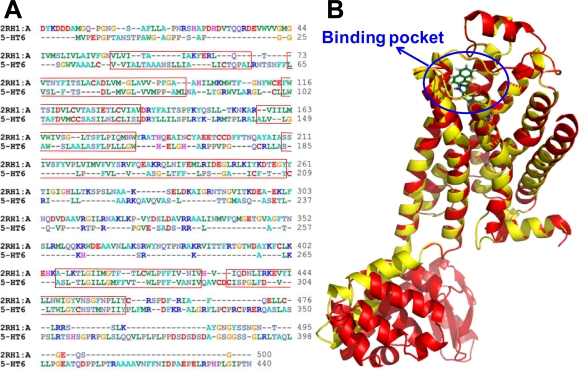
Homology modeling results. (**A**) Sequence alignment of 2RH1 (Chain A) and the 5-HT_6_ receptor model, where the important transmembrane domains (TM1-TM7) are marked in red frame; (**B**) Superposition of the template protein 2RH1 chain A (red ribbon) and the 5-HT_6_ receptor model structure (yellow ribbon) from homology modeling. Compound 198 with bottle-green stick is binding into the pocket.

Figure 4 shows the structural superposition of the 5-HT_6_ receptor homology model to the X-ray crystal structure of the template molecule (PDB ID: 2RH1). This is a high resolution crystal structure of human β_2_-adrenergic G protein-coupled receptor which has been successfully applied to homology modeling study for both CB1 and CB2 receptors [34]. Seemingly, the sequence identity between 5-HT_6_ receptor model and template 2RH1 is not so high (29% obtained from the automated mode report from [35]). However, previous reports have illustrated the key active site are focus on the transmembrane domains [36–38]. Thus, we computed the sequence identity in the important transmembrane domains (TM1-TM7), which is about 54% (87/162), arriving at the normal criterion that a sequence identity higher than 30% could be used to predict the protein structure [39]. As shown in Figure 4A, the seven transmembrane domains are marked in red frame. As seen in Figure 4B, compound 198 is docked into the pocket of the 5-HT_6_ receptor, and the template protein 2RH1 chain A (red ribbon) are well superposed with the 5-HT_6_ receptor model structure (yellow ribbon) obtained from the homology modeling, especially, around the binding pocket area. All these results certify that the 5-HT_6_ protein model we developed is reasonable.

### 2.4. Docking Results

**Figure 5.**
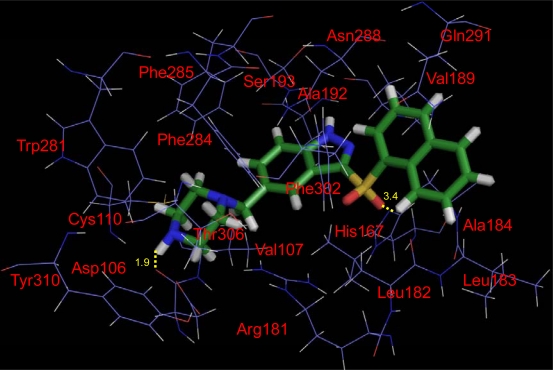
The binding result of compound 198 with 5-HT_6_. The ligand are colored in bottle-green and key amino acid residues in red labels around compound 198 within 4 Å. H-bonds are shown in yellow dash lines.

Docking studies were carried out on the dataset to find the optimal conformation of the ligand in the binding pocket of the 5-HT_6_ protein in Figure 5. Herein, compound 198 was selected as an example to demonstrate the docking model. The hydrogen bonds between the ligand and the receptor are shown as the yellow dash lines, and some significant residues near compound 198 with 4 Å are labeled in red.

It can be noticed that in this figure, several hydrophobic residues (such as Cys110, Tyr310, Trp281, Thr306, Val107 and Phe284) are able to form a large hydrophobic pocket, accommodating a large substituent in order to increase the activity. This is consistent with our previous steric-favored contour map at these regions shown in Figures 2A and 3A. The previous reports also illustrated that the amino acid residues such as Trp281 and Phe284 played a central role in ligand binding [36,38]. Another hydrophobic cavity can be found between Ser193, Ala192, Phe284, Phe285, Asn288, Phe302 and Leu182, which acts like a clamp so as to make the indazole fixed. This conclusion can be supported by above hydrophobic-favored white contour map in Figure 3C. Also, it can be noticed that the hydrophobic residues (Phe284, Phe285) in TM6 can interact with benzene ring well on the indazole. This is consistent with the previous investigation [38]. On the right of indazole, one open hydrophobic pocket is formed among Leu183, Ala184, Val189 and Gln291, which are able to interact with the naphthyl ring well. In addition, two important H-bond interactions are formed, where one is between the distal –NH of the piperazine ring and the carbonyl group of Asp106 with a length of 1.9 Å. This amino acid residue has been identified as conserved aspartate (Asp106 on TM3) by site-directed mutagenesis studies with rat 5-HT_6_ receptors [37]. This is a high degree of homology between rat and human 5-HT_6_ receptors. Several modeling investigations also implicated a role for Asp106 for interaction with the ligands [36,38]. Another hydrogen bond can be found between the –SO_2_ group and the charged His167 residue with H-bond length of 3.4 Å. Furthermore, these observations in this work are in agreement with the previous results [24,31]. Thus, above consistence of the contour maps and docking results further support the rationality of our observations. But for further validation of these conclusions, MD simulation was carried out continuously.

### 2.5. MD Simulations

**Figure 6.**
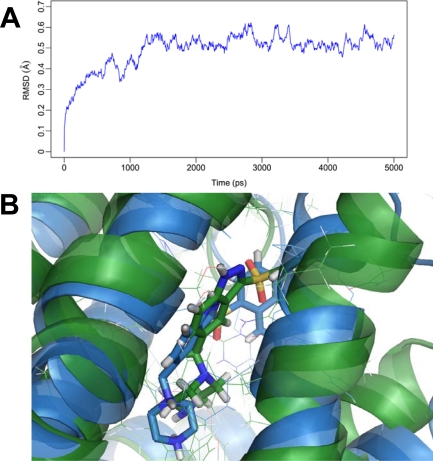
MD results. (**A**) RMSD of the docked complex versus MD simulation time in the MD-simulated structure; (**B**) Superimposition of backbone atoms in the average structure of MD simulations (light-blue) and the initial docking structure (bottle-green) for compound 198-5-HT_6_ complex. Herein, Compound 198 is represented as carbon-chain in bottle-green for the docking complex and light-blue for the average complex, respectively.

In order to examine the stability of the docking solution, presently, MD simulations lasting 5 ns was performed to the docked complex structure of 5-HT_6_ with the most potent 198, obtaining a dynamic picture of the conformational changes that occur in an aqueous solution, with main emphasis to explore the conformational change that takes place in the compound 198 and 5-HT_6_ receptor. After 1.5 ns, the RMSD of the complex tends to be stable (at about 0.55 Å) and retains this value throughout the simulation (Figure 6A), indicating stabilization of the complex. A superimposition of the average structure of 5 ns and the initial docked structure is shown in Figure 6B, where the bottle-green ribbon represents the initial structure for the docked complex, the light-blue ribbon represents the average structure of MD simulations, with compound 198 represented as carbon-chain in bottle-green for the initial complex and carbon-chain in light-blue for the average complex, respectively. It is noted that there is no significant difference between the average structure extracted from MD simulations and the docked model of the complex, proving the rationality and validity of the docking model.

## 3. Material and Experimental Methods

### 3.1. Dataset and Biological Activity

A dataset of 223 5-HT_6_ receptor ligands with *K*_i_ experimental values were collected from the continued efforts of Wyeth Research [14–21]. The corresponding p*K*_i_ (−log*K*_i_) values which were converted from the binding affinity *K*_i_ values, were used as the dependent variables in the 3D-QSAR analyses. The whole dataset was divided into a training set (184 molecules) for the generation of the QSAR models and a test se (39 molecules) for the final external validation of the systems. The selection principle of the test set was to assure that both their p*K*_i_ values and structural diversity are randomly but representative of the range of the whole set. Table S2 (Supporting Information) lists all the structures and biological values (p*K*_i_) of the dataset, where the representative skeletons and p*K*_i_ values are depicted in Table 2.

### 3.2. Molecular Alignment

**Figure 7.**
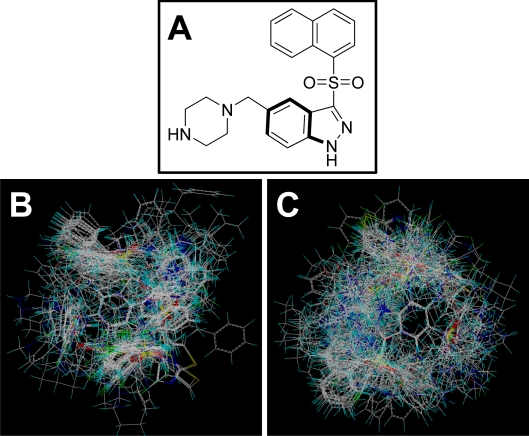
Alignment of all molecules in the dataset. (**A**) Compound 198 is used as the template. The common substructure (shown in bold) for the alignment of all molecules; (**B**) Ligand-based; and (**C**) receptor-based alignment of all the compounds.

All molecular calculations were performed using the SYBYL6.9 molecular modeling software package (Tripos Associates, St. Louis, MO). The geometry optimizations of all compounds were carried out by using the TRIPOS force field with the Gasteiger Hückel charges, and repeated minimization was performed using Powell conjugated gradient algorithm until the root-mean-square deviation (RMSD) of 0.001 kcal/mol was achieved. Molecular alignment of compounds is a crucial step for the successful development of 3D-QSAR models [40]. In this process, the most potent compound 198 was chosen as a template to fit the remaining training and test set of compounds. Thereafter, all compounds finally minimized with the lowest energy in the dataset were aligned to a common substructure by substructure-based alignment method using the “align database” command in SYBYL. Figure 7A depicts the common substructure for the alignment which is marked in bold. Figure 7B, C show the resulting ligand-based and receptor-based alignment model, respectively.

### 3.3. CoMFA and CoMSIA Field Calculation

A 3D cubic lattice with grid spacing of 2.0 Å in x, y and z directions was finally generated to encompass the aligned molecules to derive the CoMFA and CoMSIA descriptor fields. In CoMFA, a sp^3^ carbon probe atom which has a charge of +1.0 (default probe atom in SYBYL) and energy cut-off values of 30 kcal/mol, was placed at each lattice point to compute the descriptors of steric and electrostatic fields. The CoMFA steric and electrostatic fields generated were automatically scaled using the CoMFA-STD method in SYBYL. In CoMSIA, the similarity indices calculated at regularly spaced grid intervals for the pre-aligned molecules, were derived with the same lattice box implemented in SYBYL as that used for the CoMFA calculations. In addition to steric and electrostatic fields, hydrophobic, and hydrogen-bond donor and acceptor descriptors were calculated with the same lattice box of a regularly placed grid of 2.0 Å, employing a probe atom with radius 1.0 Å, charge +1.0, and hydrophobicity +1.0 CoMSIA similarity indices (*A_F_*) for a molecule *j* with atom *i* at a grid point *q* were calculated by Equation (1):

(1) $$A_{F,k}^{q}(j)\;=\;-\;\sum \omega_{\mathrm{probe},k}\omega_{\mathrm{ik}}e^{-\alpha r_{\mathrm{iq}}^{2}}$$

where *k* represents the steric, electrostatic, hydrophobic, or hydrogen-bond donor or acceptor descriptor. *ω_probe,k_* is the probe atom with radius 1.0 Å, charge +1.0, hydrophobicity +1.0, H-bond donating +1.0, H-bond accepting +1.0; *ω_ik_* is the actual value of the physicochemical property *k* of atom *i; r_iq_* is the mutual distance between the probe atom at grid point *q* and atom *i* of the test molecule. The attenuation factor α was set to 0.3.

### 3.4. 3D-QSAR Model Calculation and Validation

For deducing the 3D-QSAR models, the CoMFA and CoMSIA descriptors served as the independent variables and p*K*_i_ values as the dependent variables in the PLS regression analysis. PLS is an extension of multiple regression analysis in which the original variables are replaced by a small set of their linear combinations [41]. As a statistical approach, PLS generalizes and combines those features obtained from the principal component analysis and multiple regressions. It is particularly useful for prediction of a set of dependent variables from a large set of independent variables, especially when the matrix of predictors has more variables than observations.

To evaluate the reliability of the models generated from the PLS analysis, cross-validation analysis was accomplished with the leave-one-out (LOO) methodology where one compound was moved away from the dataset and its activity was predicted by the model derived from the rest of the dataset. The predictive value of the models was evaluated first by LOO cross-validation process, and the number of components resulting in the highest cross-validated *q*^2^ and lowest standard error of estimates (*SEE*) was then determined as the optimum number of principal components (*OPN*) in the final PLS analyses. To evaluate the predictive power of the CoMFA and CoMSIA models, the predictive *r*^2^_pred_ based on the test set molecules was calculated using equation (2):

(2) $$r_{\mathrm{pred}}^{2}\;=\;1-\mathrm{PRESS}\;/\;\mathrm{SD}$$

where *SD* denotes the sum of squared deviation between the biological activities of the test set molecules and the mean activity of the training set molecules, *PRESS* represents the sum of squared deviations between the experimental and predicted activities of the test molecules, respectively. Finally, the CoMFA and CoMSIA results were graphically represented by field contour maps, where the coefficients were generated using the field type “Stdev*Coeff”.

### 3.5. Homology Modeling

When the experimental 3D-structure of the protein is not available, homology modeling is a powerful tool with homologous proteins whose 3D structures are known. In the present study, due to the unavailability of human 5-HT_6_ 3D-structures, homology modeling process was employed to predict the protein structure from the target amino acid sequence obtained from the National Center for Biotechnology Information database [42]. The homology model of 5-HT_6_ was built in Automated Mode and the amino acid sequence of 5-HT_6_ was submitted to SWISS-MODEL server [35]. The template protein (PDB code: 2RH1 chain A, obtained from the Protein Data Bank [43], a high resolution (2.4 Å) crystal structure of human β_2_-adrenergic G protein-coupled receptor [44], was employed to generate the 3D protein structure.

### 3.6. Molecular Docking

To throw light on the interaction and illustrate the accurate binding model for the active site of 5-HT_6_ with ligands, molecular docking analysis was carried out by using the Surflex Dock implemented in SYBYL. The protein preparation and refinement utility in SYBYL was used to further develop the resulting homology protein structure. Then different conformers of all 223 ligands were docked into the binding site. During the docking process, critical parameters for generating the binding pocket, *i.e.*, the protomol_bloat which can be used to inflate the protomol and include nearby crevices and the protomol_threshold which is a factor determining how much the protomol can be buried in the protein, were set to 0.5 and 1, respectively.

### 3.7. Molecular Dynamics Simulations

The MD simulations were performed with GROMACS software package [45] using the GROMOS96 force field [46]. The molecular topology file for the ligand in protein was generated by the program PRODRG 2.5 [47,48]. The simulation cell was a cubic periodic box with a side length of 112.52 Å, and the minimum distance between the protein and box walls was set to larger than 10 Å. Herein, in order to neutralize the total charge, 17 chloridions were placed randomly in the box. The total number of the atoms of the simulation system was 138230 including the protein complexes and waters. The remaining box volume was filled using the simple point charge (SPC) water [49]. Prior to the simulation, an energy minimization was applied to the full system without constraints using the steepest descent integrator for 8854 steps, then the system was equilibrated via a 200 ps MD simulation at 300 K. Finally, a 5 ns simulation was performed with a time step of 2 fs. During MD simulation, the standard parameters and main calculation methods were set as follows: The model used NPT ensemble at 300 K with periodic boundary conditions, the temperature was kept constant by the Berendsen thermostat, the value of the isothermal compressibility was set to 4.5 × 10^−5^ bar^−1^ while the pressure was maintained at 1 bar using the Parrinello-Rahman scheme [50], the electrostatic interactions were calculated using the particle mesh Ewald method [51,52], the cut-off distances for the calculation of Coulomb and van der Waals interactions were 1.0 and 1.4 nm, respectively. All the MD simulations lasted 5 ns to ensure that the whole systems were stable.

## 4. Conclusions

In the present article, a large dataset including 223 5-HT_6_ receptor ligands has been estimated for the purpose of developing 3D-QSAR models based on both the ligand- and receptor-based superimpositions. Statistically significant models have been derived with two 3D-QSAR methods of CoMFA and CoMSIA on the basis of the database alignment method. These two approaches produce equally good models expressed in terms of several rigorous evaluation criteria such as *q*^2^ and *r*^2^_pred_ for both internal and external data sets. Graphical interpretation of the optimal results, provided by the CoMFA and CoMSIA analyses, brings to light important structural features that could be responsible for the low- or high-binding activity to 5-HT_6_ receptor. In addition, a good consistency between the CoMFA and CoMSIA contour maps, molecular docking and molecular dynamics simulations proves the reliability and robustness of the developed models. The contour maps for the series of 5-HT_6_ ligands reveal some information about the modification of these compounds. Taking the most potent ligand 198 as an example, the introduction of a large and H-bond donor substituent at the 5-position on the indazole will increase the potency. The presence of an H-bond acceptor (such as –SO_2_) at 3-position of the indazole plays a positive role in enhancing the activity of the ligand. In addition, the especially important amino acid residue of Asp106 is essential to interact with ligands. We hope the developed models could provide an insight into some instructions for further synthesis of highly potent 5-HT_6_ receptor ligands.

## Supplementary Information



## Figures and Tables

**Table 1.** Summary of ligand-based and receptor-based 3D-QSAR models ^a^.

**PLS Statistics**	**Ligand-based model**		**Receptor-based model**	

	**CoMFA**	**CoMSIA**	**CoMFA**	**CoMSIA**
*q*^2^	0.50	0.53	0.45	0.47
*OPN*	7	7	5	5
*r*^2^_ncv_	0.85	0.81	0.78	0.75
*SEE*	0.27	0.30	0.33	0.35
*F*	127.90	104.23	123.45	104.26
*r*^2^_pred_	0.71	0.76	0.59	0.62
Contribution Steric	0.42	0.18	0.42	0.16
Electrostatic	0.46	0.33	0.42	0.26
Hydrophobic	-	0.24		0.25
H-bond acceptor	-	0.12		0.16
CIC2	0.07	0.07	0.11	0.12
BEHv2	0.05	0.06	0.05	0.05

a , *q*^2^: Cross-validated correlation coefficient after the leave-one-out procedure; *r*^2^_ncv_: Non-cross-validated correlation coefficient; *SEE*: standard error of estimate; *F: F* statistical value; *r*^2^_pred_: Predicted correlation coefficient for the test set of compounds; *OPN*: optimal number of principal components; CIC2: neighborhood symmetry of 2-order; BEHv2: the highest eigenvalue n. 2 of Burden matrix weighted by atomic van der Waals volumes Burden eigenvalues.

**Table 2.** Representative structures and binding affinity values p*K*_i_ (M) of the dataset.

**No.**	**Structure**	**p*K*_i_**	**No.**	**Structure**	**p*K*_i_**
1	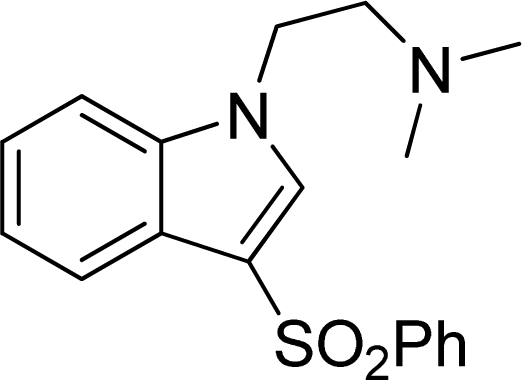	7.70	99	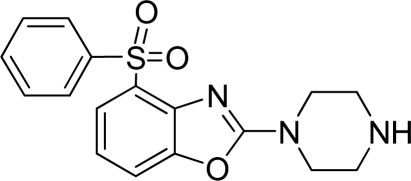	7.39
2	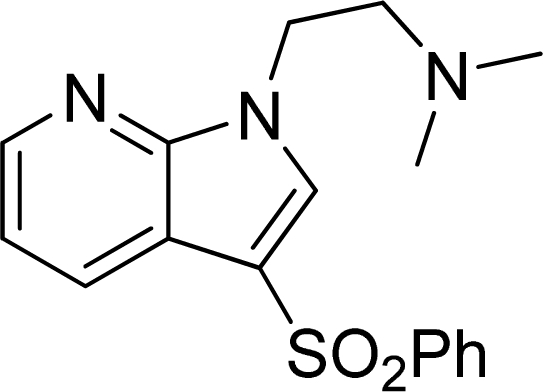	7.64	100 *	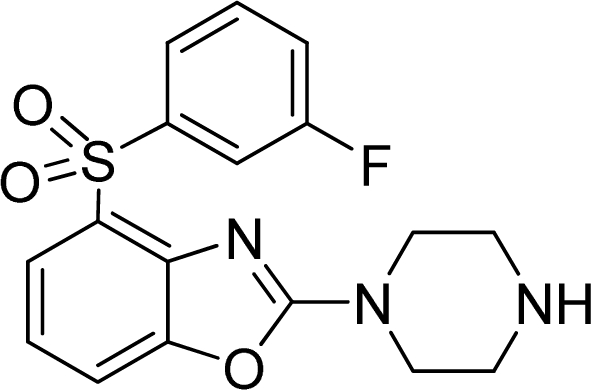	7.27
3	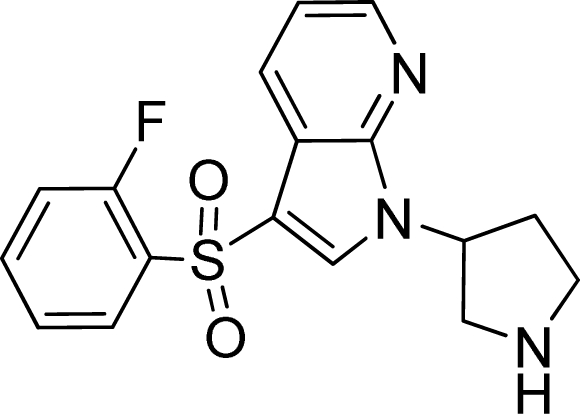	8.70	121	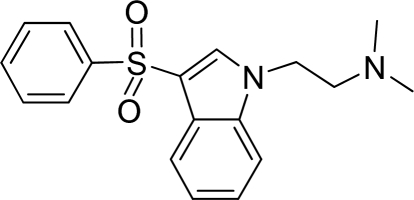	7.70
27	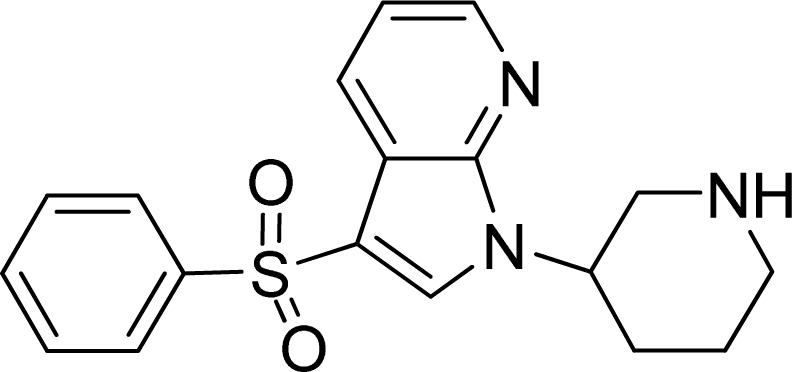	8.31	132	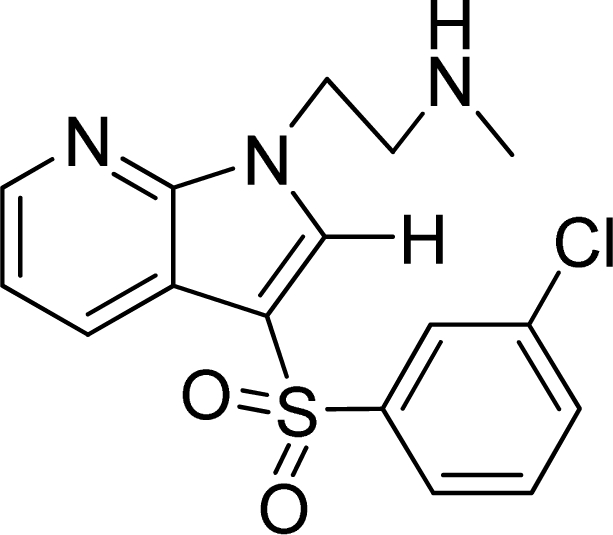	9.52
55	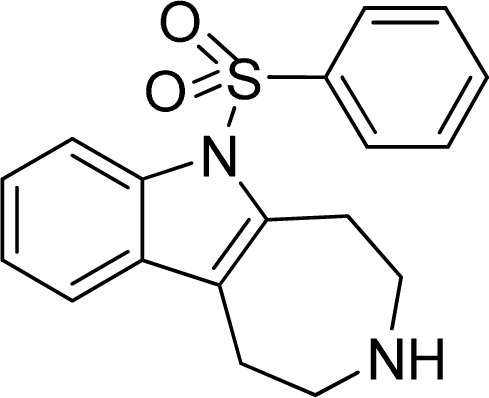	6.71	149	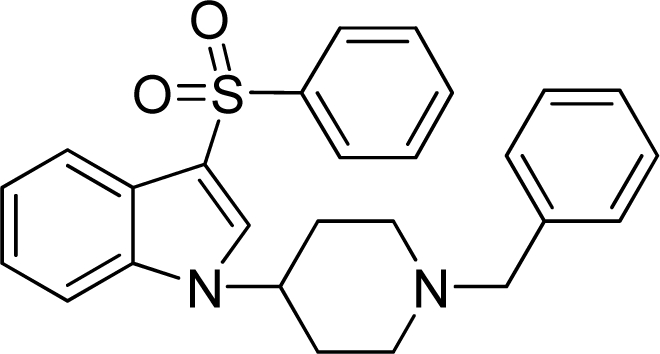	7.31
56 *	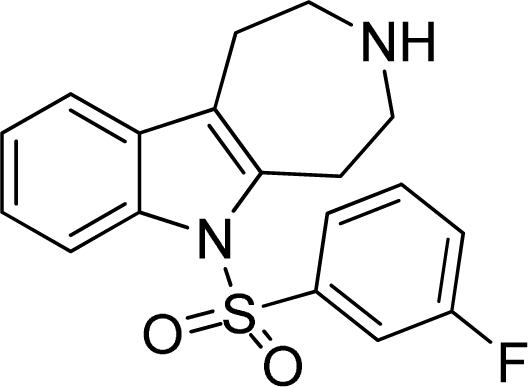	7.48	179	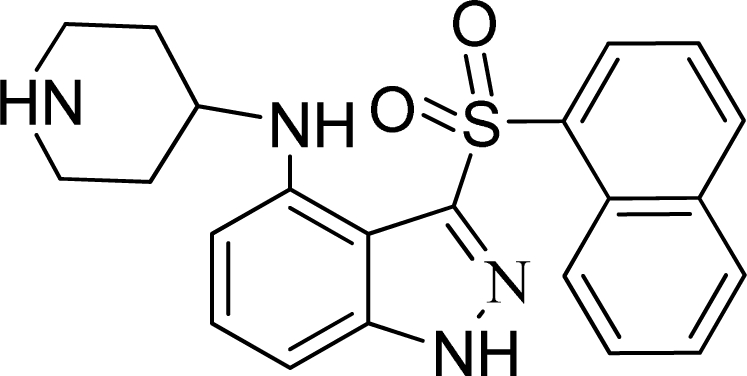	8.55
74	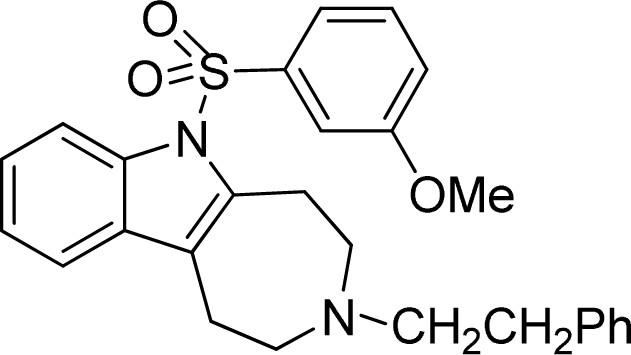	6.30	198	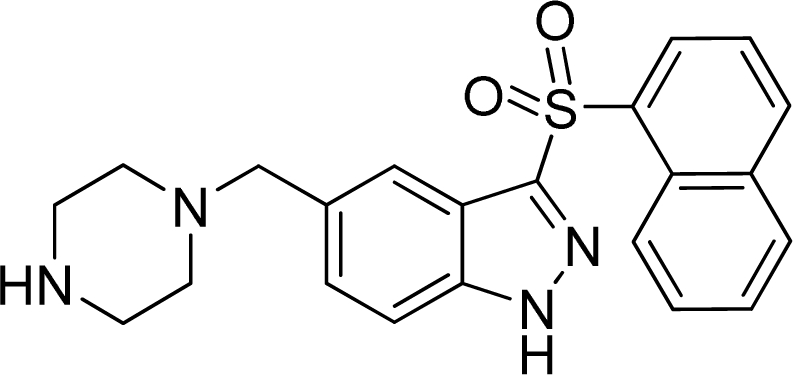	9.62

* Compounds belonged to the test set.

## References

[1] Pullagurla M, Bondareva T, Young R, Glennon RA (2004). Modulation of the stimulus effects of (+) amphetamine by the 5-HT_6_ antagonist MS-245. Pharmacol. Biochem. Behav.

[2] Bromidge SM, Brown AM, Clarke SE, Dodgson K, Gager T, Grassam HL, Jeffrey PM, Joiner GF, King FD, Middlemiss DN (1999). 5-chloro-*N*-(4-methoxy-3-piperazin-1-ylphenyl)-3-methyl-2-benzothiophenesulfon-amide (SB-271046): A potent, selective, and orally bioavailable 5-HT_6_ receptor antagonist. J. Med. Chem.

[3] Meneses A (2001). Effects of the 5-HT_6_ receptor antagonist Ro 04-6790 on learning consolidation. Behav. Brain Res.

[4] Hamon M, Doucet E, Lefèvre K, Miquel M-C, Lanfumey L, Insausti R, Frechilla D, Del Rio J, Vergé D (1999). Antibodies and antisense oligonucleotide for probing the distribution and putative functions of central 5-HT_6_ receptors. Neuropsychopharmacology.

[5] Mitchell ES, Neumaier JF (2005). 5-HT_6_ receptors: A novel target for cognitive enhancement. Pharmacol. Ther.

[6] Millan MJ (2003). The neurobiology and control of anxious states. Prog. Neurobiol.

[7] Schechter LE, Ring RH, Beyer CE, Hughes ZA, Khawaja X, Malberg JE, Rosenzweig-Lipson S (2005). Innovative approaches for the development of antidepressant drugs: Current and future strategies. Neurotherapeutics.

[8] Wesolowska A (2010). Potential role of the 5-HT_6_ receptor in depression and anxiety: An overview of preclinical data. Pharmacol. Rep.

[9] Kohen R, Metcalf MA, Khan N, Druck T, Huebner K, Lachowicz JE, Meltzer HY, Sibley DR, Roth BL, Hamblin MW (1996). Cloning, characterization, and chromosomal localization of a human 5-HT_6_ serotonin receptor. J. Neurochem.

[10] Monsma FJ, Shen Y, Ward RP, Hamblin MW, Sibley DR (1993). Cloning and expression of a novel serotonin receptor with high affinity for tricyclic psychotropic drugs. Mol. Pharmacol.

[11] Plassat JL, Amlaiky N, Hen R (1993). Molecular cloning of a mammalian serotonin receptor that activates adenylate cyclase. Mol. Pharmacol.

[12] Glennon RA, Lee M, Rangisetty JB, Dukat M, Roth BL, Savage JE, McBride A, Rauser L, Hufeisen S, Lee DKH (2000). 2-substituted tryptamines: agents with selectivity for 5-HT_6_ serotonin receptors. J. Med. Chem.

[13] Pullagurla MR, Dukat M, Setola V, Roth B, Glennon RA (2003). *N*_1_-benzenesulfonylgramine and *N*_1_-benzenesulfonylskatole: novel 5-HT_6_ receptor ligand templates. Bioorg. Med. Chem. Lett.

[14] Elokdah H, Li D, McFarlane G, Bernotas RC, Robichaud AJ, Magolda RL, Zhang GM, Smith D, Schechter LE (2007). Novel 1-(azacyclyl)-3-arylsulfonyl-1*H*-pyrrolo[2,3-*b*]pyridines as 5-HT_6_ agonists and antagonists. Bioorg. Med. Chem.

[15] Liu KG, Lo JR, Comery TA, Zhang GM, Zhang JY, Kowal DM, Smith DL, Di L, Kerns EH, Schechter LE (2008). A regiospecific synthesis of a series of 1-sulfonyl azepinoindoles as potent 5-HT_6_ ligands. Bioorg. Med. Chem. Lett.

[16] Liu KG, Lo JR, Comery TA, Zhang GM, Zhang JY, Kowal DM, Smith DL, Di L, Kerns EH, Schechter LE (2009). 1-sulfonylindazoles as potent and selective 5-HT_6_ ligands. Bioorg. Med. Chem. Lett.

[17] Liu KG, Lo JR, Comery TA, Zhang GM, Zhang JY, Kowal DM, Smith DL, Di L, Kerns EH, Schechter LE (2009). Identification of a novel series of 3-piperidinyl-5-sulfonylindazoles as potent 5-HT_6_ ligands. Bioorg. Med. Chem. Lett.

[18] Liu KG, Lo JR, Comery TA, Zhang GM, Zhang JY, Kowal DM, Smith DL, Di L, Kerns EH, Schechter LE (2009). Identification of a series of benzoxazoles as potent 5-HT_6_ ligands. Bioorg. Med. Chem. Lett.

[19] Bernotas RC, Lenicek S, Antane S, Cole DC, Harrison BL, Robichaud AJ, Zhang GM, Smith D, Platt B, Lin Q (2009). Novel 1-aminoethyl-3-arylsulfonyl-1*H*-pyrrolo[2,3-*b*] pyridines are potent 5-HT_6_ agonists. Bioorg. Med. Chem.

[20] Bernotas RC, Antane S, Shenoy R, Le V-D, Chen P, Harrison BL, Robichaud AJ, Zhang GM, Smith D, Schechter LE (2010). 3-(arylsulfonyl)-1-(azacyclyl)-1H-indoles are 5-HT_6_ receptor modulators. Bioorg. Med. Chem. Lett.

[21] Liu KG, Robichaud AJ, Greenfield AA, Lo JR, Grosanu C, Mattes JF, Cai Y, Zhang GM, Zhang JY, Kowal DM (2011). Identification of 3-sulfonylindazole derivatives as potent and selective 5-HT_6_ antagonists. Bioorg. Med. Chem.

[22] Sleight AJ, Boess FG, Bös M, Levet-Trafit B, Riemer C, Bourson A (1998). Characterization of Ro 04-6790 and Ro 63-0563: potent and selective antagonists at human and rat 5-HT_6_ receptors. Br. J. Pharmacol.

[23] Boess FG, Riemer C, Bös M, Bentley J, Bourson A, Sleight AJ (1998). The 5-hydroxytryptamine_6_ receptor-selective radioligand [^3^H]Ro 63–0563 labels 5-hydroxytryptamine receptor binding sites in rat and porcine striatum. Mol. Pharmacol.

[24] Holenz J, Pauwels PJ, Díaz JL, Mercè R, Codony X, Buschmann H (2006). Medicinal chemistry strategies to 5-HT_6_ receptor ligands as potential cognitive enhancers and antiobesity agents. Drug Discov. Today.

[25] Russell MGN, Dias R (2002). Memories are made of this (perhaps): A review of serotonin 5-HT_6_ receptor ligands and their biological functions. Curr. Top. Med. Chem.

[26] Muthas D, Sabnis YA, Lundborg M, Karlén A (2008). Is it possible to increase hit rates in structure-based virtual screening by pharmacophore filtering? An investigation of the advantages and pitfalls of post-filtering. J. Mol. Graph. Model.

[27] Xie H-Z, Li L-L, Ren J-X, Zou J, Yang L, Wei Y-Q, Yang S-Y (2009). Pharmacophore modeling study based on known spleen tyrosine kinase inhibitors together with virtual screening for identifying novel inhibitors. Bioorg. Med. Chem. Lett.

[28] Alzate-Morales JH, Vergara-Jaque A, Caballero J (2010). Computational study on the interaction of N1 substituted pyrazole derivatives with B-Raf kinase: An unusual water wire hydrogen-bond network and novel interactions at the entrance of the active site. J. Chem. Inf. Model.

[29] Villacañas O, Pérez JJ, Rubio-Martínez J (2002). Structural analysis of the inhibition of Cdk4 and Cdk6 by p16 (INK4a) through molecular dynamics simulations. J. Biomol. Struct. Dyn.

[30] Doddareddy MR, Cho YS, Koh HY, Pae AN (2004). CoMFA and CoMSIA 3D QSAR analysis on *N*_1_-arylsulfonylindole compounds as 5-HT_6_ antagonists. Bioorg. Med. Chem.

[31] López-Rodríguez ML, Benhamú B, de la Fuente T, Sanz A, Pardo L, Campillo M (2005). A three-dimensional pharmacophore model for 5-hydroxytryptamine_6_ (5-HT_6_) receptor antagonists. J. Med. Chem.

[32] Goodarzi M, Freitas MP, Ghasemi N (2010). QSAR studies of bioactivities of 1-(azacyclyl)-3-arylsulfonyl-1H-pyrrolo[2,3-*b*]pyridines as 5-HT_6_ receptor ligands using physicochemical descriptors and MLR and ANN-modeling. Eur. J. Med. Chem.

[33] Sharma BK, Singh P, Sarbhai K, Prabhakar YS (2010). A quantitative structure-activity relationship study on serotonin 5-HT_6_ receptor ligands: indolyl and piperidinyl sulphonamides. SAR QSAR Environ. Res.

[34] Durdagi S, Papadopoulos MG, Zoumpoulakis PG, Koukoulitsa C, Mavromoustakos T (2010). A computational study on cannabinoid receptors and potent bioactive cannabinoid ligands: Homology modeling, docking, de novo drug design and molecular dynamics analysis. Mol. Divers.

[35] SWISS-MODEL http://swissmodel.expasy.org/.

[36] Pullagurla MR, Westkaemper RB, Glennon RA (2004). Possible differences in modes of agonist and antagonist binding at human 5-HT_6_ receptors. Bioorg. Med. Chem. Lett.

[37] Hirst WD, Abrahamsen B, Blaney FE, Calver AR, Aloj L, Price GW, Medhurst AD (2003). Differences in the central nervous system distribution and pharmacology of the mouse 5-hydroxytryptamine-6 receptor compared with rat and human receptors investigated by radioligand binding, site-directed mutagenesis, and molecular modeling. Mol. Pharmacol.

[38] Dukat M, Mosier PD, Kolanos R, Roth BL, Glennon RA (2008). Binding of serotonin and N_1_-benzenesulfonyltryptamine-related analogs at human 5-HT_6_ serotonin receptors: Receptor modeling studies. J. Med. Chem.

[39] Hillisch A, Pineda LF, Hilgenfeld R (2004). Utility of homology models in the drug discovery process. Drug Discov. Today.

[40] AbdulHameed MDM, Hamza A, Liu JJ, Zhan CG (2008). Combined 3D-QSAR modeling and molecular docking study on indolinone derivatives as inhibitors of 3-phosphoinositide-dependent protein kinase-1. J. Chem. Inf. Model.

[41] Böhm M, Stürzebecher J, Klebe G (1999). Three-dimensional quantitative structure-activity relationship analyses using comparative molecular field analysis and comparative molecular similarity indices analysis to elucidate selectivity differences of inhibitors binding to trypsin, thrombin, and factor Xa. J. Med. Chem.

[42] NCBI http://www.ncbi.nlm.nih.gov/.

[43] RCSB PDB http://www.rcsb.org/pdb/home/home.do/.

[44] Cherezov V, Rosenbaum DM, Hanson MA, Rasmussen SGF, Thian FS, Kobilka TS, Choi H-J, Kuhn P, Weis WI, Kobilka BK (2007). High-resolution crystal structure of an engineered human β_2_-adrenergic G protein-coupled receptor. Science.

[45] Berendsen HJC, van der Spoel D, van Drunen R (1995). GROMACS: A message-passing parallel molecular dynamics implementation. Comput. Phys. Commun.

[46] Lindahl E, Hess B, van der Spoel D (2001). GROMACS 3.0: a package for molecular simulation and trajectory analysis. J. Mol. Model.

[47] Aalten DMF, Bywater R, Findlay JBC, Hendlich M, Hooft RWW, Vriend G (1996). PRODRG, a program for generating molecular topologies and unique molecular descriptors from coordinates of small molecules. J. Comput. Aided Mol. Des.

[48] Schuttelkopf AW, van Aalten DMF (2004). PRODRG: A tool for high-throughput crystallography of protein-ligand complexes. Acta Crystallogr.

[49] Berendsen HJC, Postma JPM, van Gunsteren WF, Hermans J, Pullman B (1981). Intermolecular Forces.

[50] Parrinello M, Rahman A (1981). Polymorphic transitions in single crystals: A new molecular dynamics method. J. Appl. Phys.

[51] Essmann U, Perera L, Berkowitz M, Darden T, Lee H, Pedersen L (1995). A smooth particle mesh Ewald method. J. Chem. Phys.

[52] Lin JH, Perryman AL, Schames JR, McCammon JA (2002). Computational drug design accommodating receptor flexibility: The relaxed complex scheme. J. Am. Chem. Soc.

