# Supplementary material for: Investigation of the Structure Requirement for 5-HT6 Binding Affinity of Arylsulfonyl Derivatives: A Computational Study
Source: Int J Mol Sci. 2011 Aug 8;12(8):5011–30. doi: 10.3390/ijms12085011 (PMC3179148; doi:10.3390/ijms12085011)
Supplement: Supplementary file 1 [file ijms-12-05011-s001.pdf]

Supplementary Information

Ming Hao, Yan Li *, Hanqing Li and Shuwei Zhang

Department of Materials Science and Chemical Engineering, Dalian University of Technology, Dalian 116023, Liaoning, China; E-Mails: dluthm@yeah.net (M.H.); lihanqing00@gmail.com (H.L.); zswei@dlut.edu.cn (S.Z.)

* Author to whom correspondence should be addressed; E-Mail: yanli@dlut.edu.cn; Tel.: +86-411-84986062; Fax: +86-411-84986063.

Received: 8 July 2011; in revised form: 19 July 2011 / Accepted: 29 July / Published: 8 August 2011

Abstract: 5-HT_6 receptor has been implicated in a series of diseases including anxiety, depression, schizophrenia and cognitive dysfunctions. 5-HT_6 ligands have been reported to play a significant role in the treatment for central nervous system (CNS) diseases. Presently, a large series of 223 5-HT_6 ligands were studied using a combinational method by 3D-QSAR, molecular docking and molecular dynamics calculations for further improvement of potency. The optimal 3D models exhibit satisfying statistical results with r^2_{ncv}, q^2 values of 0.85 and 0.50 for CoMFA, 0.81 and 0.53 for CoMSIA, respectively. Their predictive powers were validated by external test set, showing r^2_{pred} of 0.71 and 0.76. The contour maps also provide a visual representation of contributions of steric, electrostatic, hydrophobic and hydrogen bond fields as well as the prospective binding models. In addition, the agreement between 3D-QSAR, molecular docking and molecular dynamics simulation proves the rationality of the developed models. These results, we hope, may be helpful in designing novel and potential 5-HT_6 ligands.

Keywords: 5-HT_6; 3D-QSAR; CoMFA; CoMSIA; molecular dynamics

Table S1. The observed and predicted pK_i values of the 3D-QSAR models.

No.	Observed	Ligand-based Models	
		CoMFA	CoMSIA
1	7.70	7.61	7.86
2	7.64	7.69	7.78
3	8.70	8.62	8.60
4*	8.57	8.62	8.61
5	8.07	8.34	8.43
6	8.82	8.71	8.68
7	8.26	8.41	8.52
8*	8.89	8.76	8.62
9	8.37	8.54	8.61
10	8.89	8.86	8.62
11	8.60	8.24	8.26
12*	8.25	8.67	8.54
13	8.85	8.75	8.58
14	8.30	8.26	8.40
15	8.66	8.71	8.59
16*	8.82	8.58	8.67
17	8.38	8.37	8.35
18	9.05	8.76	8.74
19	7.96	8.08	8.13
20*	8.08	8.46	8.45
21	8.48	8.49	8.49
22	9.00	8.85	8.77
23	8.24	8.40	8.74
24*	9.00	9.16	9.04
25	9.10	9.14	9.14
26	8.85	8.71	8.92
27	8.31	8.10	8.26
28*	8.44	7.76	7.98
29	8.43	8.49	8.55
30	8.89	8.49	8.54
31	8.12	8.23	8.42
32*	7.94	7.86	8.18
33	7.47	7.79	8.06
34	7.95	7.76	7.94
35	8.34	8.62	8.50
36	7.92	7.75	8.20
37	8.68	8.72	8.38
38	8.42	8.75	8.61
39	8.70	8.66	8.52

Table S1. *Cont.*

No.	Observed	Ligand-based Models	
		CoMFA	CoMSIA
40*	8.62	8.68	8.56
41	8.33	8.49	8.43
42	8.36	8.41	8.31
43	8.47	8.49	8.32
44	8.03	8.50	8.53
45	8.21	8.46	8.44
46	8.41	8.27	8.30
47	7.87	8.19	8.21
48	8.92	8.79	8.68
49	8.72	8.61	8.52
50	8.49	8.46	8.46
51	9.15	9.03	9.26
52	8.70	8.65	8.72
53	9.15	9.04	9.18
54	8.77	9.00	9.18
55	6.71	6.92	6.92
56*	7.48	7.36	7.22
57	7.64	7.34	7.29
58	7.72	7.50	7.18
59	7.39	7.32	7.22
60*	7.74	7.44	7.33
61	7.17	7.32	7.36
62	7.54	7.57	7.43
63	7.05	6.96	7.11
64*	7.34	7.37	7.35
65	7.62	7.74	7.47
66	7.72	7.76	7.57
67	7.48	7.59	7.55
68	7.92	7.57	7.16
69	7.72	7.73	7.63
70	7.30	7.36	7.42
71	6.91	6.85	6.99
72*	7.07	6.75	6.90
73	6.50	7.14	7.00
74	6.30	6.27	6.15
75	6.49	6.62	6.69
76*	6.60	6.42	6.54
77	7.92	7.68	7.69
78	8.10	7.76	8.14
79	7.30	7.23	7.36

Table S1. *Cont.*

No.	Observed	Ligand-based Models	
		CoMFA	CoMSIA
80	9.30	8.86	8.77
81	8.92	8.89	8.84
82	8.74	8.73	8.76
83	8.68	8.70	8.73
84*	7.82	8.36	8.53
85	8.64	8.86	8.57
86	8.89	9.01	8.81
87	7.74	7.59	7.54
88*	7.26	7.02	7.22
89	7.03	7.31	7.70
90	7.72	7.32	7.56
91	7.30	7.55	7.65
92	7.18	7.20	7.33
93	7.34	7.00	7.33
94	7.85	7.60	7.53
95	7.32	7.28	7.23
96*	6.91	6.91	6.89
97	8.42	8.07	8.00
98	8.02	7.79	7.77
99	7.39	7.49	7.39
100*	7.27	7.58	7.56
101	7.19	7.36	7.40
102	7.51	7.62	7.47
103	7.66	7.87	7.92
104*	7.66	7.65	7.32
105	7.48	7.18	7.18
106	7.60	7.96	7.98
107	7.89	7.97	7.81
108	8.51	8.09	7.95
109	7.28	6.92	7.17
110	6.71	6.93	7.07
111	8.15	7.99	8.03
112*	8.01	7.90	7.82
113	7.92	7.73	7.64
114	7.44	7.46	7.50
115	7.89	7.59	7.47
116	7.06	7.35	7.38
117	6.75	6.94	7.00
118	6.90	6.91	6.89
119	7.14	7.31	7.23

Table S1. *Cont.*

No.	Observed	Ligand-based Models	
		CoMFA	CoMSIA
120*	7.10	7.38	7.21
121	7.70	7.66	7.83
122	7.60	7.70	7.96
123	7.64	7.74	7.81
124*	8.26	8.27	8.24
125	8.38	8.67	8.29
126	8.33	8.27	7.87
127	8.32	8.15	8.17
128	9.00	8.50	8.48
129	8.33	8.76	8.72
130	7.06	7.45	7.59
131	8.64	8.14	8.21
132	9.52	8.65	8.59
133	8.70	8.86	8.75
134	7.35	7.56	7.75
135	7.35	7.75	7.86
136*	7.66	8.44	8.38
137	8.68	8.65	8.75
138	8.68	8.89	8.67
139	8.37	7.98	8.19
140*	8.54	8.67	8.70
141	7.93	7.88	8.13
142	8.05	7.96	8.09
143	9.00	8.87	8.91
144*	8.80	7.96	8.27
145	7.82	8.06	8.02
146	8.48	8.15	8.16
147	9.05	8.85	8.78
148*	9.05	9.52	9.29
149	7.31	7.10	7.61
150	7.04	7.08	7.46
151	6.91	7.23	7.30
152*	7.89	8.00	7.76
153	8.14	7.64	7.46
154	8.22	8.01	7.94
155	7.97	7.94	8.07
156	7.17	7.82	7.93
157	7.80	7.45	7.51
158	8.40	7.99	7.91
159	7.68	8.23	8.25

Table S1. *Cont.*

No.	Observed	Ligand-based Models	
		CoMFA	CoMSIA
160	8.15	8.18	8.21
161	8.25	8.45	8.33
162	6.95	6.86	6.77
163	8.30	8.18	8.04
164*	8.33	8.28	8.21
165	8.46	8.26	8.25
166	7.74	7.65	7.70
167	8.02	8.14	8.19
168*	8.30	8.25	8.23
169	7.74	7.70	7.73
170	7.82	8.41	8.37
171	7.14	7.98	7.94
172*	8.49	8.42	8.34
173	8.89	8.55	8.36
174	8.34	8.15	7.87
175	7.63	8.10	8.23
176*	8.22	8.18	8.21
177	8.82	8.93	8.93
178	8.55	9.04	8.97
179	8.55	8.57	8.64
180*	8.38	8.83	8.79
181	8.02	8.05	7.95
182	8.17	8.17	8.24
183	8.85	9.12	9.07
184*	8.37	8.29	8.32
185	9.15	8.63	8.77
186	8.77	8.32	8.45
187	8.46	8.54	8.38
188*	7.77	7.84	7.89
189	7.28	7.17	7.10
190	7.24	7.24	7.17
191	7.36	7.49	7.63
192*	7.77	7.88	7.79
193	7.52	7.57	7.75
194	7.18	7.55	7.61
195	7.77	7.64	7.65
196*	7.92	8.64	8.59
197	7.85	8.16	7.94
198	9.62	9.07	9.11
199	8.82	8.85	8.96

Table S1. *Cont.*

No.	Observed	Ligand-based Models	
		CoMFA	CoMSIA
200	8.96	8.85	8.99
201	7.62	7.95	7.74
202	7.36	7.20	7.11
203	7.19	7.12	6.87
204	7.92	7.79	7.54
205	7.51	7.58	7.56
206	8.47	8.49	8.50
207	8.32	8.32	8.07
208*	7.57	7.83	7.65
209	7.62	7.84	7.80
210	7.55	7.90	7.78
211	8.01	8.00	7.92
212	8.54	8.33	8.16
213	6.97	6.81	7.12
214	8.51	8.26	8.29
215	8.17	8.44	8.44
216*	9.00	8.65	8.57
217	9.10	8.89	8.77
218	8.20	8.42	8.36
219	8.64	8.55	8.46
220*	9.00	8.43	8.47
221	8.82	8.77	8.39
222	8.01	8.56	8.44
223	9.22	9.26	9.33

*, test set.

Table S2. The structures of molecules with skeleton type A in the dataset.

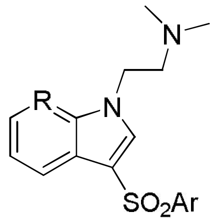

No.	Ar	R	CIC2	BEHv2	pK_i	Ref[a]
1	Ph	C	1.62	3.80	7.70	[14]
2	Ph	N	1.28	3.79	7.64	[14]

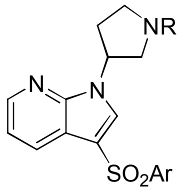

No.	Ar	R	CIC2	BEHv2	p*K*i	Refa
3	2-FPh	H	0.74	3.79	8.70	[14]
4*	3-FPh	H	0.69	3.79	8.57	[14]
5	4-FPh	H	0.79	3.79	8.07	[14]
6	3-ClPh	H	0.69	3.80	8.82	[14]
7	4-ClPh	H	0.79	3.80	8.26	[14]
8*	3-BrPh	H	0.69	3.81	8.89	[14]
9	4-BrPh	H	0.79	3.81	8.37	[14]
10	3-CF3Ph	H	0.75	3.82	8.89	[14]
11	Ph	Me	1.00	3.79	8.60	[14]
12*	2-FPh	Me	0.80	3.80	8.25	[14]
13	3-FPh	Me	0.75	3.80	8.85	[14]
14	4-FPh	Me	0.84	3.80	8.30	[14]
15	2-ClPh	Me	0.80	3.80	8.66	[14]
16*	3-ClPh	Me	0.75	3.80	8.82	[14]
17	4-ClPh	Me	0.84	3.80	8.38	[14]
18	3-BrPh	Me	0.75	3.81	9.05	[14]
19	Ph	Et	1.10	3.79	7.96	[14]
20*	3-FPh	Et	0.87	3.80	8.08	[14]
21	3-ClPh	Et	0.87	3.80	8.48	[14]
22	5-Cl-thien-2-yl	H	0.54	3.78	9.00	[14]
23	8-Quinolyl	H	0.94	3.93	8.24	[14]
24*	6-Cl-imidazo[2,1-*b*]thiazol-5-yl	H	0.40	3.90	9.00	[14]
25	6-Cl-imidazo[2,1-*b*]thiazol-5-yl	Me	0.48	3.90	9.10	[14]
26	6-Cl-imidazo[2,1-*b*]thiazol-5-yl	Et	0.61	3.90	8.85	[14]

Table S2. *Cont.*

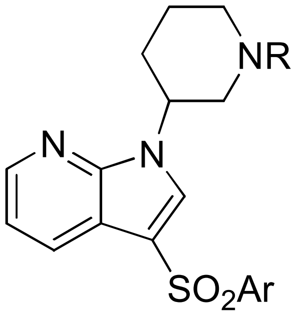

No.	Ar	R	CIC2	BEHv2	pK_i	Ref[a]
27	Ph	H	1.03	3.79	8.31	[14]
28*	Ph	Et	1.16	3.79	8.44	[14]
29	2-ClPh	H	0.83	3.80	8.43	[14]
30	3-ClPh	H	0.78	3.80	8.89	[14]
31	4-ClPh	H	0.87	3.80	8.12	[14]
32*	4-ClPh	Me	0.92	3.80	7.94	[14]
33	4-ClPh	Et	1.02	3.80	7.47	[14]
34	4-ClPh	*i*-Pr	1.10	3.80	7.95	[14]
35	3-CF_3Ph	H	0.83	3.82	8.34	[14]
36	4-CF_3Ph	H	0.92	3.82	7.92	[14]
37	3-CF_3Ph	Me	0.88	3.82	8.68	[14]
38	3-BrPh	H	0.78	3.81	8.42	[14]
39	2-FPh	H	0.83	3.79	8.70	[14]
40*	3-FPh	H	0.78	3.79	8.62	[14]
41	3-FPh	Me	0.83	3.79	8.33	[14]
42	3-FPh	Et	0.93	3.79	8.36	[14]
43	3-FPh	*i*-Pr	1.02	3.79	8.47	[14]
44	3-BrPh	*i*-Pr	1.02	3.81	8.03	[14]
45	3,5-diClPh	H	0.83	3.81	8.21	[14]
46	3,5-diClPh	Me	0.88	3.81	8.41	[14]
47	3,5-diClPh	Et	0.98	3.81	7.87	[14]
48	5-Cl-thien-2-yl	H	0.65	3.78	8.92	[14]
49	5-Cl-thien-2-yl	Me	0.72	3.78	8.72	[14]
50	5-Cl-thien-2-yl	Et	0.83	3.78	8.49	[14]
51	6-Cl-imidazo[2,1-*b*]thiazol-5-yl	H	0.51	3.90	9.15	[14]
52	6-Cl-imidazo[2,1-*b*]thiazol-5-yl	Me	0.58	3.90	8.70	[14]
53	6-Cl-imidazo[2,1-*b*]thiazol-5-yl	Et	0.70	3.90	9.15	[14]
54	6-Cl-imidazo[2,1-*b*]thiazol-5-yl	*i*-Pr	0.80	3.90	8.77	[14]

Table S2. *Cont.*

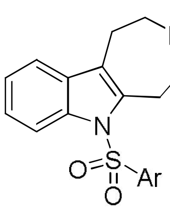

No.	Ar	R	CIC2	BEHv2	pK_i	Ref[a]
55	Ph	H	1.52	3.81	6.71	[15]
56*	3-F-Ph	H	1.19	3.81	7.48	[15]
57	4-F-Ph	H	1.22	3.81	7.64	[15]
58	2-Cl-Ph	H	1.27	3.82	7.72	[15]
59	3-Cl-Ph	H	1.19	3.82	7.39	[15]
60*	4-Cl-Ph	H	1.22	3.82	7.74	[15]
61	3-Me-Ph	H	1.22	3.84	7.17	[15]
62	4-Me-Ph	H	1.24	3.84	7.54	[15]
63	3-CF_3-Ph	H	1.22	3.84	7.05	[15]
64*	4-CF_3-Ph	H	1.24	3.84	7.34	[15]
65	5-Cl-Naph	H	1.40	3.98	7.62	[15]
66	2-MeO-Ph	H	1.26	3.82	7.72	[15]
67	4-MeO-Ph	H	1.22	3.82	7.48	[15]
68	3-MeO-Ph	H	1.19	3.82	7.92	[15]
69	3-MeO-Ph	Me	1.21	3.82	7.72	[15]
70	3-MeO-Ph	Et	1.29	3.82	7.30	[15]
71	3-MeO-Ph	*n*-Pr	1.26	3.82	6.91	[15]
72*	3-MeO-Ph	*i*-Pr	1.32	3.82	7.07	[15]
73	3-MeO-Ph	Bn	1.59	3.82	6.50	[15]
74	3-MeO-Ph	$PhCH_2CH_2$	1.81	3.82	6.30	[15]
75	3-MeO-Ph	*c*-Pentyl	1.41	3.82	6.49	[15]
76*	3-MeO-Ph	*c*-Hexyl	1.53	3.82	6.60	[15]

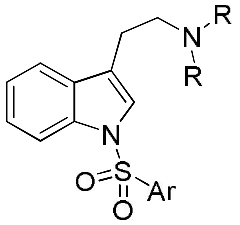

No.	Ar	R	CIC2	BEHv2	pK_i	Ref[a]
77	Ph	Et	1.61	3.81	7.92	[15]

Table S2. *Cont.*

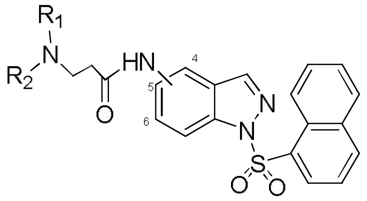

No	Position	R_1	R_2	CIC2	BEHv2	pK_i	Ref[a]
78	4	H	H	1.17	3.95	8.10	[16]
79	5	H	H	1.10	3.94	7.30	[16]
80	6	H	H	1.17	3.94	9.30	[16]
81	6	CH_3	H	1.16	3.94	8.92	[16]
82	6	CH_3	CH_3	1.34	3.94	8.74	[16]
83	6	CH_3CH_2	CH_3CH_2	1.47	3.94	8.68	[16]
84*	6	$-CH_2CH_2CH_2CH_2CH_2-$		1.44	3.94	7.82	[16]

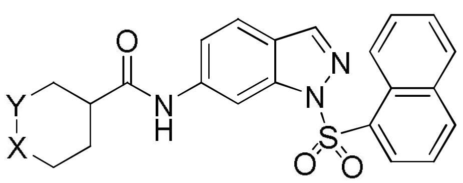

No.	X	Y	CIC2	BEHv2	pK_i	Ref[a]
85	NH	CH_2	1.28	3.94	8.64	[16]
86	CH_2	NH	1.21	3.94	8.89	[16]

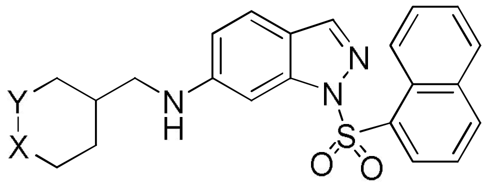

No.	X	Y	CIC2	BEHv2	pK_i	Ref[a]
87	NH	CH_2	1.40	3.94	7.74	[16]

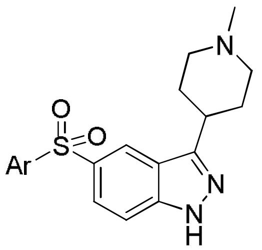

No.	Ar	CIC2	BEHv2	pK_i	Ref[a]
88*	Ph	1.35	3.82	7.26	[17]
89	3-F-Ph	1.34	3.94	7.03	[17]
90	3-Cl-Ph	1.10	3.83	7.72	[17]
91	3-Me-Ph	1.13	3.85	7.30	[17]

Table S2. *Cont.*

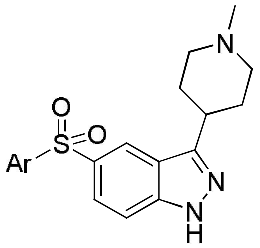

No.	Ar	CIC2	BEHv2	pK_i	Ref[a]
92	4-F-Ph	1.20	3.83	7.18	[17]
93	4-CF_3-Ph	1.22	3.84	7.34	[17]
94	4-*i*Pr-Ph	1.36	3.86	7.85	[17]
95	4-CF_3O-Ph	1.20	3.83	7.32	[17]
96*	4-MeO-Ph	1.20	3.83	6.91	[17]
97	1-Naph	1.38	3.96	8.42	[17]
98	2-Naph	1.44	3.96	8.02	[17]

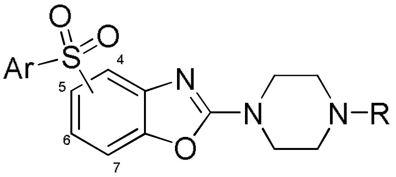

No.	Position	Ar	R	CIC2	BEHv2	pK_i	Ref[a]
99	4	Ph	H	1.63	3.78	7.39	[18]
100*	4	3-F-Ph	H	1.31	3.78	7.27	[18]
101	4	4-F-Ph	H	1.38	3.78	7.19	[18]
102	4	3-Cl-Ph	H	1.31	3.78	7.51	[18]
103	4	4-*i*Pr-Ph	H	1.48	3.82	7.66	[18]
104*	4	3-CF_3-Ph	H	1.33	3.80	7.66	[18]
105	4	4-CF_3-Ph	H	1.39	3.80	7.48	[18]
106	4	3-MeO-Ph	H	1.30	3.79	7.60	[18]
107	4	2,5-diCl-Ph	H	1.21	3.79	7.89	[18]
108	4	1-Naph	H	1.66	3.92	8.51	[18]
109	5	1-Naph	H	1.59	3.91	7.28	[18]
110	6	1-Naph	H	1.59	3.91	6.71	[18]
111	7	1-Naph	H	1.66	3.92	8.15	[18]
112*	4	1-Naph	Me	1.66	3.92	8.01	[18]
113	4	1-Naph	Et	1.74	3.92	7.92	[18]
114	4	1-Naph	n-Pr	1.68	3.92	7.44	[18]
115	4	1-Naph	*i*-Pr	1.71	3.92	7.89	[18]
116	4	1-Naph	*n*-Bu	1.69	3.92	7.06	[18]
117	4	1-Naph	*i*-Bu	1.78	3.92	6.75	[18]
118	4	1-Naph	$Ph(CH_2)_3$	1.97	3.92	6.90	[18]
119	4	1-Naph	*c*-Bu	1.68	3.92	7.14	[18]
120*	4	1-Naph	*c*-Pen	1.77	3.92	7.10	[18]

Table S2. *Cont.*

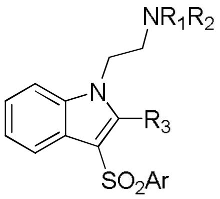

No.	Ar	R_1	R_2	R_3	CIC2	BEHv2	pK_i	Ref[a]
121	Ph	Me	Me	H	1.62	3.80	7.70	[19]
122	Ph	H	H	H	1.46	3.80	7.60	[19]

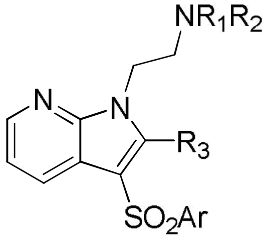

No.	Ar	R_1	R_2	R_3	CIC2	BEHv2	pK_i	Ref[a]
123	Ph	Me	Me	H	1.28	3.79	7.64	[19]
124*	Ph	H	H	H	1.07	3.79	8.26	[19]
125	Ph	H	H	Me	1.11	3.79	8.38	[19]
126	Ph	Me	Me	Me	1.30	3.79	8.33	[19]
127	3-FPh	Me	Me	H	1.02	3.79	8.32	[19]
128	3-FPh	Me	H	H	0.78	3.79	9.00	[19]
129	3-FPh	H	H	H	0.76	3.79	8.33	[19]
130	3-FPh	-$(CH_2)_4$-		H	1.16	3.79	7.06	[19]
131	3-ClPh	Me	Me	H	1.02	3.80	8.64	[19]
132	3-ClPh	Me	H	H	0.78	3.80	9.52	[19]
133	3-ClPh	H	H	H	0.76	3.80	8.70	[19]
134	3-ClPh	-$(CH_2)_4$-		H	1.16	3.80	7.35	[19]
135	4-FPh	Me	Me	H	1.12	3.79	7.35	[19]
136*	4-FPh	H	H	H	0.88	3.79	7.66	[19]
137	2-CF_3Ph	H	H	H	0.88	3.82	8.68	[19]
138	3-CF_3Ph	H	H	H	0.83	3.81	8.68	[19]
139	3,5-DiClPh	Me	Me	H	1.07	3.80	8.37	[19]
140*	3,5-DiClPh	H	H	H	0.82	3.80	8.54	[19]
141	2,5-DiClPh	Me	Me	H	1.03	3.80	7.93	[19]
142	2,6-DiClPh	Me	Me	H	1.07	3.80	8.05	[19]
143	1-Naphthyl	Me	Me	H	1.43	3.94	9.00	[19]
144*	1-Naphthyl	H	H	H	1.27	3.94	8.80	[19]
145	2-Thienyl	Me	Me	H	0.91	3.77	7.82	[19]
146	5-Cl-thien-2-yl	Me	Me	H	0.91	3.78	8.48	[19]
147	6-Cl-imidazo[2,1-*b*][1,3]thiazo-5-yl	Me	Me	H	0.75	3.89	9.05	[19]
148*	6-Cl-imidazo[2,1-*b*][1,3]thiazo-5-yl	Me	H	H	0.48	3.89	9.05	[19]

Table S2. *Cont.*

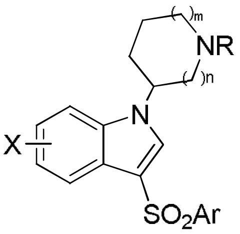

No.	Azacycle	Ar	X	R	CIC2	BEHv2	pK_i	Ref[a]
149	4-Piperidinyl	Ph	H	CH_2Ph	1.85	3.81	7.31	[20]
150	4-Piperidinyl	Ph	H	H	1.46	3.81	7.04	[20]
151	3-Piperidinyl	Ph	H	CH_2Ph	1.78	3.81	6.91	[20]
152*	3-Piperidinyl	Ph	H	H	1.37	3.81	7.89	[20]
153	3-Piperidinyl	Ph	H	Et	1.45	3.81	8.14	[20]
154	3-Piperidinyl	3-FPh	H	H	1.06	3.81	8.22	[20]
155	3-Piperidinyl	3-FPh	5-OMe	H	0.88	3.81	7.97	[20]
156	3-Piperidinyl	3-FPh	5-OMe	Me	0.93	3.81	7.17	[20]
157	3-Piperidinyl	3-FPh	5-OMe	Et	1.01	3.81	7.80	[20]
158	3-Piperidinyl	3-FPh	5-F	H	0.99	3.81	8.40	[20]
159	3-Piperidinyl	1-Naphthyl	H	H	1.51	3.97	7.68	[20]
160	3-Piperidinyl	1-Naphthyl	H	Me	1.52	3.97	8.15	[20]
161	3-Piperidinyl	8-Quinolinyl	H	H	1.17	3.94	8.25	[20]
162	3-Pyrrolidinyl	Ph	H	CH_2Ph	1.77	3.81	6.95	[20]
163	3-Pyrrolidinyl	Ph	H	H	1.32	3.81	8.30	[20]
164*	3-Pyrrolidinyl	3-FPh	H	H	0.99	3.81	8.33	[20]
165	3-Pyrrolidinyl	3-FPh	H	Me	1.03	3.81	8.46	[20]
166	3-Pyrrolidinyl	3-FPh	4-F	H	0.92	3.81	7.74	[20]
167	3-Pyrrolidinyl	3-FPh	5-F	H	0.92	3.81	8.02	[20]
168*	3-Pyrrolidinyl	3-FPh	5-F	Me	0.96	3.81	8.30	[20]
169	3-Pyrrolidinyl	3-FPh	6-F	H	0.92	3.81	7.74	[20]
170	3-Pyrrolidinyl	3-FPh	5-Cl	H	0.77	3.81	7.82	[20]
171	3-Pyrrolidinyl	3-FPh	6-Cl	H	0.77	3.81	7.14	[20]
172*	3-Pyrrolidinyl	3-ClPh	H	H	0.99	3.81	8.49	[20]
173	3-Pyrrolidinyl	3-ClPh	H	Me	1.03	3.81	8.89	[20]
174	3-Pyrrolidinyl	3-ClPh	6-OMe	H	0.81	3.82	8.34	[20]
175	3-Pyrrolidinyl	1-Naphthyl	H	H	1.48	3.97	7.63	[20]
176*	3-Pyrrolidinyl	1-Naphthyl	H	Me	1.49	3.97	8.22	[20]
177	3-Pyrrolidinyl	8-Quinolinyl	H	H	1.09	3.94	8.82	[20]
178	3-Pyrrolidinyl	8-Quinolinyl	H	Me	1.12	3.94	8.55	[20]

Table S2. *Cont.*

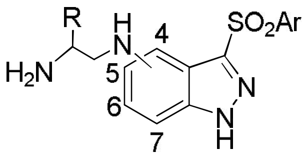

(A)

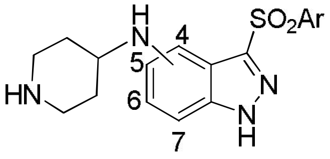

(B)

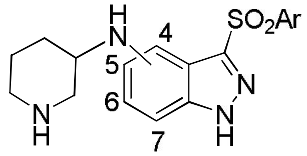

(C)

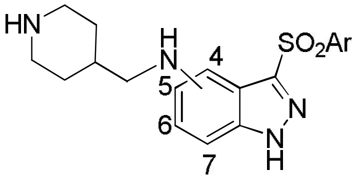

(D)

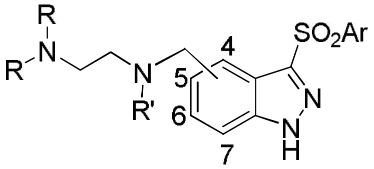

(E)

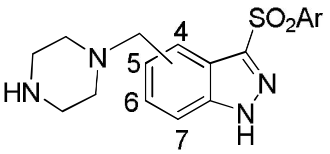

(F)

No.	Class	Position	R	R'	Ar	CIC2	BEHv2	pK_i	Ref[a]
179	B	4	-	-	1-Naph	1.33	3.95	8.55	[21]
180*	C	4	-	-	1-Naph	1.25	3.95	8.38	[21]
181	D	4	-	-	1-Naph	1.40	3.95	8.02	[21]
182	A	5	H	-	1-Naph	1.24	3.94	8.17	[21]
183	A	5	Me	-	1-Naph	1.14	3.94	8.85	[21]
184*	A	5	(*R*)-*i*Pr	-	1-Naph	1.25	3.94	8.37	[21]
185	B	5	-	-	1-Naph	1.27	3.94	9.15	[21]
186	C	5	-	-	1-Naph	1.19	3.94	8.77	[21]
187	D	5	-	-	1-Naph	1.34	3.94	8.46	[21]
188*	A	6	H	-	1-Naph	1.32	3.94	7.77	[21]
189	B	6	-	-	1-Naph	1.33	3.94	7.28	[21]
190	C	6	-	-	1-Naph	1.25	3.94	7.24	[21]
191	D	6	-	-	1-Naph	1.40	3.94	7.36	[21]
192*	A	7	H	-	1-Naph	1.39	3.94	7.77	[21]
193	B	7	-	-	1-Naph	1.40	3.95	7.52	[21]
194	C	7	-	-	1-Naph	1.32	3.95	7.18	[21]
195	D	7	-	-	1-Naph	1.46	3.95	7.77	[21]
196*	E	5	H	H	1-Naph	1.21	3.95	7.92	[21]
197	E	5	CH_3	CH_3	1-Naph	1.61	3.95	7.85	[21]
198	F	5	-	-	1-Naph	1.47	3.95	9.62	[21]

Table S2. *Cont.*

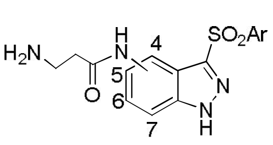

(A)

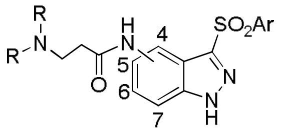

(B)

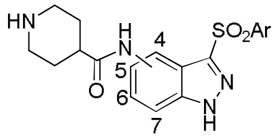

(C)

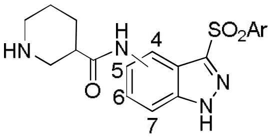

(D)

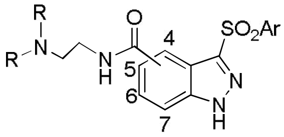

(E)

No	Class	Position	R	Ar	CIC2	BEHv2	pK_i	Ref[a]
199	A	5	-	1-Naph	1.10	3.94	8.82	[21]
200	C	5	-	1-Naph	1.22	3.94	8.96	[21]
201	A	6	-	1-Naph	1.17	3.94	7.62	[21]
202	B	6	CH_3	1-Naph	1.34	3.94	7.36	[21]
203	B	6	CH_3CH_2	1-Naph	1.47	3.94	7.19	[21]
204	B	6	$-(CH_2)_5-$	1-Naph	1.44	3.94	7.92	[21]
205	C	6	-	1-Naph	1.28	3.94	7.51	[21]
206	A	7	-	1-Naph	1.24	3.95	8.47	[21]
207	B	7	CH_3CH_2	1-Naph	1.40	3.95	8.32	[21]
208*	B	7	CH_3CH_2	1-Naph	1.52	3.95	7.57	[21]
209	B	7	$-(CH_2)_5-$	1-Naph	1.50	3.95	7.62	[21]
210	C	7	-	1-Naph	1.34	3.95	7.55	[21]
211	D	7	-	1-Naph	1.27	3.95	8.01	[21]
212	E	5	CH_3	1-Naph	1.35	3.95	8.54	[21]
213	E	5	$-(CH_2)_5-$	1-Naph	1.50	3.95	6.97	[21]

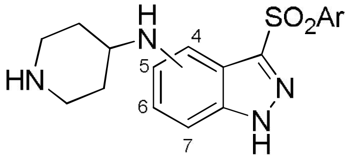

No.	Position	Ar	CIC2	BEHv2	pK_i	Ref[a]
214	5	Ph	1.17	3.80	8.51	[21]
215	5	3-F-Ph	0.93	3.80	8.17	[21]
216*	5	3-Cl-Ph	0.93	3.81	9.00	[21]
217	5	3-Me-Ph	0.97	3.83	9.10	[21]
218	5	4-F-Ph	1.01	3.80	8.20	[21]
219	5	4-Cl-Ph	1.01	3.81	8.64	[21]
220*	5	4-*i*Pr-Ph	1.17	3.84	9.00	[21]
221	5	$4-CF_3-Ph$	1.05	3.83	8.82	[21]
222	5	4-MeO-Ph	1.03	3.81	8.01	[21]
223	5	2-Naph	1.25	3.95	9.22	[21]

*, test set; [a], from the corresponding reference.

© 2011 by the authors; licensee MDPI, Basel, Switzerland. This article is an open access article distributed under the terms and conditions of the Creative Commons Attribution license (http://creativecommons.org/licenses/by/3.0/).
